# Pharmaceutical Incompatibility of Lubricating Gel Formulation Reduces Antibacterial Activity of Chlorhexidine Gluconate: In Vitro Study in Northern Thailand

**DOI:** 10.3390/ijerph191912285

**Published:** 2022-09-27

**Authors:** Thanawat Pattananandecha, Sasithorn Sirilun, Sutasinee Apichai, Teerapat Ouirungroj, Phisit Uirungroj, Fumihiko Ogata, Naohito Kawasaki, Chalermpong Saenjum

**Affiliations:** 1 Center of Excellence for Innovation in Analytical Science and Technology for Biodiversity-Based Economic and Society (I-ANALY-S-T_B.BES-CMU), Chiang Mai University, Chiang Mai 50200, Thailand; 2 Department of Pharmaceutical Sciences, Faculty of Pharmacy, Chiang Mai University, Chiang Mai 50200, Thailand; 3 Pose Health Care Co., Ltd., 1 Soi Ramintra 107, Ramintra Rd., Kannayao, Bangkok 10230, Thailand; 4 Faculty of Pharmacy, Kindai University, 3-4-1 Kowakae, Higashi-Osaka, Osaka 577-8502, Japan; 5 Antiaging Center, Kindai University, 3-4-1 Kowakae, Higashi-Osaka, Osaka 577-8502, Japan

**Keywords:** chlorhexidine solution, lubricating gel, pharmaceutical incompatibility, antibacterial activity, thickening agent

## Abstract

Chlorhexidine gluconate (CHG) is a cationic disinfectant. The positive charge of CHG molecules binds to phospholipid’s negative charge in bacterial cell walls, causing membrane disruption. The in vitro kinetic physical, chemical and biological incompatibilities of nine lubricating gels with 1% *w*/*v* CHG were investigated. Five containing anionic thickener, two containing nonionic thickener, and two containing cationic thickener were collected from hospitals in northern Thailand. All the anionic and nonionic lubricating gels significantly reduced (*p* < 0.05) the CHG amount after 5 min of exposure time from 12.54% to 54.99%, respectively. In contrast, the amount of CHG exposed with cationic lubricating gels was maintained. Antibacterial activity was significantly reduced to a 1.17–4.33 log10 reduction for *Staphylococcus aureus* ATCC25923 and a 1.07–3.52 log10 reduction for *Escherichia coli* ATCC25922 after 5 min exposure to all anionic and nonionic lubricating gels. In contrast, the two cationic lubricating gels maintained the antibacterial activity of the CHG solution (5.69 ± 0.14 and 5.45 ± 0.17 log10 reduction). The results suggest that anionic and nonionic thickeners in lubricating gel formulations may neutralize the positive charge and reduce the antibacterial activity of CHG, reducing its effectiveness as a disinfectant.

## 1. Introduction

Chlorhexidine gluconate (CHG) is a cationic bis-biguanide biocide with low mammalian toxicity. CHG is the most effective antiseptic and disinfectant agent, with a broad spectrum against Gram-positive bacteria, Gram-negative bacteria, and fungi [1]. The positive charge of CHG molecules binds to the negative charge of phospholipid in bacterial cell walls, causing membrane disruption [2]. CHG has bacteriostatic properties, inhibiting bacterial growth and bactericidal mechanisms of action, depending on its concentration [3], and is widely used in various concentrations from 0.12% to 4.0% in numerous topical applications [4]. CHG is available in mouthwash solutions, cosmetics, dental gels, antimicrobial handwashes, alcohol hand-rubs, toothpaste, liquid soaps, shower foams, body care products, wound care solutions, lubricants and medical ointments. Moreover, CHG has been applied in various medical tools, including dental implants, vascular catheters, urinary catheters, needleless connectors, antimicrobial dressings, and more [5,6]. 

Urinary catheter insertion is a common procedure in Intensive Care Units (ICUs) and can be a significant cause of infection in the hospital environment [7,8]. The use of CHG in the periprocedural antisepsis of urinary catheterization has contributed to decreasing urinary tract infections associated with long-term urinary catheters among patients admitted to the coronary ICU [8]. Recommendations from guidelines to prevent and control catheter-associated urinary tract infections (CAUTIs) upon urinary catheterization include minimizing catheter use, using correct insertion practices, managing appropriately, monitoring infections, and promptly removing urinary catheters [9]. During the catheterization process, CHG solution (0.1–1.0%) is a common agent used for metal cleaning before catheter insertion, followed by a urethral lubricant product before catheterization [10,11]. 

The gel consists of two components possessing both the cohesive characteristics of solids and the diffusive transport properties of liquids [12]. Polymers are used as thickening or gelling agents to provide a structural network, which is essential for preparing gels including natural polymers (gelatin, collagen, agar, pectin, and guar gum) semisynthetic polymers (cellulose derivatives including hydroxyethyl cellulose, methylcellulose, hydroxypropyl methyl cellulose, hydroxypropyl cellulose, and carboxymethyl cellulose), synthetic polymers (carbomer including carbopol-941, carbopol-940, carbopol-934, polyacrylamide, and poloxamers), and inorganic substances (bentonite and aluminum hydroxide), each having different ionic properties—neutral, anionic, and cationic [13]. Depending on the agent, gelling agents have been used at concentrations of 0.5 to 10%; carbomer is the most commonly used gelling agent [13,14]. Carbomer is the most common anionic thickener used in alcohol hand sanitizing gels and lubricating gels, allowing them to be adequately dispensed on the skin. While CHG is a cationic surfactant synthetic biguanide, its persistent activity is based on chemical deposition binding to the skin’s stratum corneum [5]. The anionic compounds can deactivate CHG’s persistent activity. Therefore, the incompatibility of anionic-based gels can significantly reduce the effectiveness of CHG residue on the skin. CHG effectiveness can also be deactivated or precipitated by anionic agents in products commonly used as emollients instantly after applying CHG [15], such as when preventing CAUTIs for periurethral cleaning or urinary catheter coating, ventilator-associated pneumonia (VAP), central line-associated bloodstream infections (CLABSIs), and short-term central venous catheters (CVC) [16,17,18,19,20,21]. Pharmaceutical incompatibility is defined as a change where an undesirable product is formed, affecting the pharmaceutical product’s safety, efficacy, appearance, or stability [22]. Physical incompatibilities are caused by interactions between two or more substances, and result in a changing color, odor, taste, viscosity, and morphology. In contrast, chemical incompatibilities lead to a change in chemical properties and are signaled by precipitation, effervescence, decomposition, and color changes [22]. The incompatibilities are more qualitative than quantitative and can be evaluated using various methods, including colorimetric and spectrophotometric evaluations.

Therefore, this study aimed at an in vitro kinetic investigation of the pharmaceutical incompatibilities, including the physical, chemical, and biological incompatibilities, of eight Thai-marketed lubricating gels frequently used in hospitals in northern Thailand and a lubricating gel formulated with CHG solution.

## 2. Materials and Methods

### 2.1. Materials

The CHG solution (20% *w*/*v* in water) was purchased from Sigma Co., Ltd. (Steinheim, Germany). All the solvents and chemicals used were purchased from Sigma Chemical Co., Ltd. (St. Louis, MO, USA) and Merck (Darmstadt, Germany). Five marketed lubricating gels containing anionic thickener (A1–A5), two marketed lubricating gels containing nonionic thickener (N1 and N2), and one marketed lubricating gel containing cationic thickener (C1) were collected from various hospitals in northern Thailand. A formulated lubricating gel containing cationic thickener (C2) was produced by the Faculty of Pharmacy, Chiang Mai University, Chiang Mai, Thailand, in collaboration with Pose healthcare Co., Ltd., Bangkok, Thailand. The types of gelling agents/thickeners in each lubricating gel sample are shown in Table 1. *Staphylococcus aureus* ATCC25923 and *Escherichia coli* ATCC25922 were purchased from the American Type Culture Collection (ATCC, Manassas, VA, USA). 

### 2.2. In Vitro Incompatibility Study

The in vitro physical and chemical incompatibilities were investigated using the modified method of Kaiser et al. [5]. Initially, 1% CHG in deionized (DI) water was freshly prepared. Then, 2 mL of 1% CHG was mixed with 1 g each of lubricating gel sample (final concentration 0.6–0.7%) for 1-, 5-, 10-, and 15-min exposure times. Then, physical incompatibility was observed by the naked eye and captured using a black and white cupboard. Additionally, the chemical incompatibility was measured for each amount of CHG using reversed-phase high-performance liquid chromatography (RP-HPLC) with the modified method of Havlíková et al. [23]. The analysis was conducted using an Agilent 1200 connected to a UV detector set at a wavelength of 239 nm. The mobile phase consisted of acetonitrile and 0.08 M sodium phosphate monobasic buffer solution containing 0.5% (*v*/*v*) triethylamine (TEA) and adjusted with phosphoric acid to pH 3.0 at a ratio of 35:65 (*v*/*v*) with isocratic elution at a flow rate of 1.0 mL/min and injection volume of 10 µL. The Symmetry Shield RP18 column (4.6 mm × 250 mm, 5 µm, Water Co., Ltd., Milford, MA, USA) was used to separate CHG, and the HPLC run time was set at 20 min. The solution collected at various exposure times was measured in triplicates. The samples were prepared by dilution with the mobile phase to a proper concentration, then filtering through a 0.45 µm syringe filter and transferring to an HPLC vial. The percent reduction in CHG was calculated according to the equation shown below.

$$Percent\;reduction=\frac{\left(CHG\;amount\;before\;exposure-CHG\;amount\;after\;exposure\right)}{CHG\;amount\;before\;exposure}\times 100\%$$

### 2.3. In Vitro Antibacterial Activity

The antibacterial activity was assessed using the modified method of Pelyuntha et al. [24]. Briefly, overnight cultures of *S. aureus* ATCC25923 and *E. coli* ATCC25922 were diluted to derive a microbial count of approximately 1.0 to 2.0 × 10^8^ CFU/mL. For the experiments, 500 µL of each reaction mixture of the lubricating gel samples and CHG solutions with 1-, 5-, 10-, and 15-min exposure times were inoculated with 500 µL of tested microbial solutions. Then, the reaction was incubated for 24 h at 37 °C under aerobic conditions. Normal saline was used as the control. To determine the bacterial number, the incubated samples were extracted in 0.85% NaCl solution. Serial dilutions were plated onto tryptic soy agar (TSA) plates and then incubated for 24 h at 37 °C. Subsequently, colonies were counted as total CFU. The log reduction was calculated according to the equation shown below.

Log reduction (24 h) = Log CFU (control or sample) (0 h) − Log CFU (sample) (24 h)

### 2.4. Statistical Analysis

All the results are expressed as a mean of three replicates ± standard deviation (SD). All the statistical analyses were performed using the SPSS Software, Version 17.00 (SPSS Inc., Chicago, IL, USA). A one-way ANOVA was used to determine any significant difference between treatments. *p* < 0.05 was considered significant, and further significance between groups was analyzed using the Duncan post hoc test.

## 3. Results

### 3.1. In Vitro Incompatibility Study

**Figure 1:**
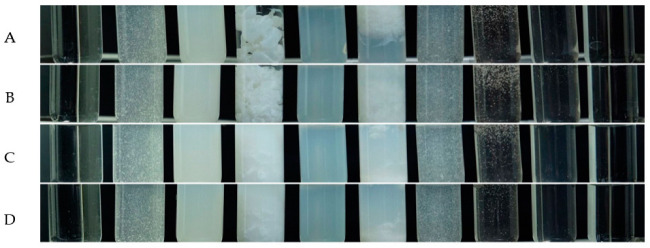
Physical incompatibility of lubricating gel samples exposed to CHG for (**A**) 1, (**B**) 5, (**C**) 10, and (**D**) 15 min, respectively.

The in vitro physical incompatibility of the 1% CHG and lubricating gel mixes at 1, 5, 10 and 15 min (A-D) is shown in Figure 1. Physical incompatibility was observed by the naked eye, involving the sedimentation or suspension of the lubricating gels in a black and white cupboard. The results show that lubricating gels containing anionic thickener A1 to A5 and lubricating gel containing nonionic thickener N1 underwent sedimentation and suspension. The lubricating gels containing nonionic thickener N2 and cationic thickener C1 and C2 formed clear solutions. In addition, incompatibility emerged after an exposure time of 1 min.

**Figure 2:**
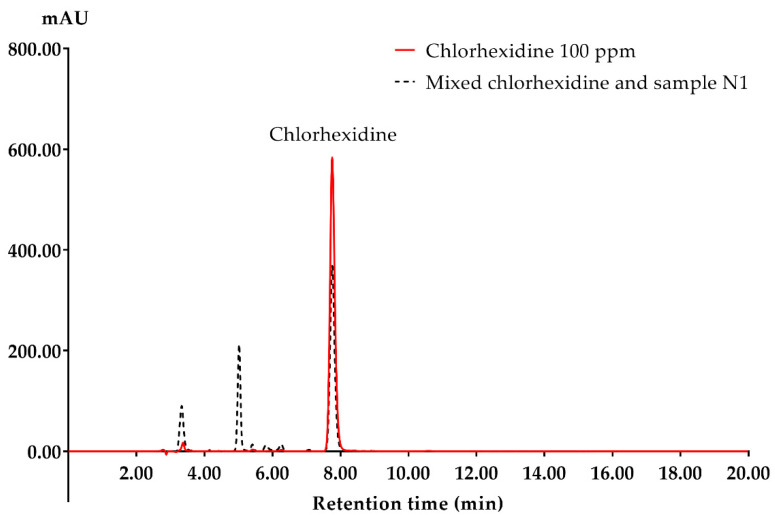
HPLC chromatogram of standard CHG and mixed CHG with lubricating gel sample N1.

**Figure 3:**
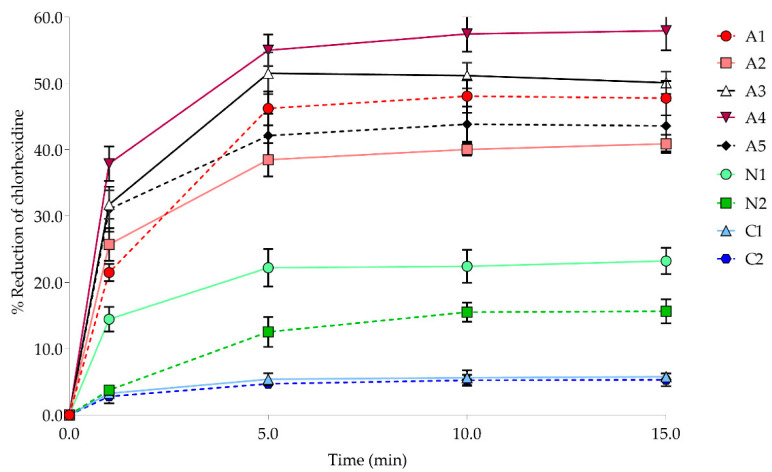
Percent reduction in CHG after exposure with lubricating gels at 1, 5, 10, and 15 min, respectively.

We then determined the amount of CHG in the mixtures using RP-HPLC and calculated the percent reduction in CHG. The HPLC chromatogram of standard CHG, and a mixture of 1% CHG and lubricating gel sample N1, is shown in Figure 2, demonstrating that CHG had a retention time of 7.75 min. The HPLC resulted in reduced CHG at different times of exposure to the tested lubricating gels, as shown in Figure 3. The amounts of CHG were significantly reduced (*p* < 0.05) after 1 and 5 min of exposure in all five lubricating gels containing anionic thickener (A1 to A5) to 21.49-54.99%, which was then maintained at 40.04% to 57.94% after 10 and 15 min of exposure. The amount of CHG after exposure to the two lubricating gels with nonionic thickener (N1 and N2) also significantly decreased (*p* < 0.05) to 3.75% to 22.21% with exposure times of 1 and 5 min, and at 10 and 15 min of exposure time, the amounts were 15.50% to 23.23%. In contrast, lubricating gels containing cationic thickeners (C1 and C2) slightly reduced the amount of CHG by 2.79 to 5.78% after exposure for 1 to 15 min. 

### 3.2. In Vitro Antibacterial Activity

**Figure 4:**
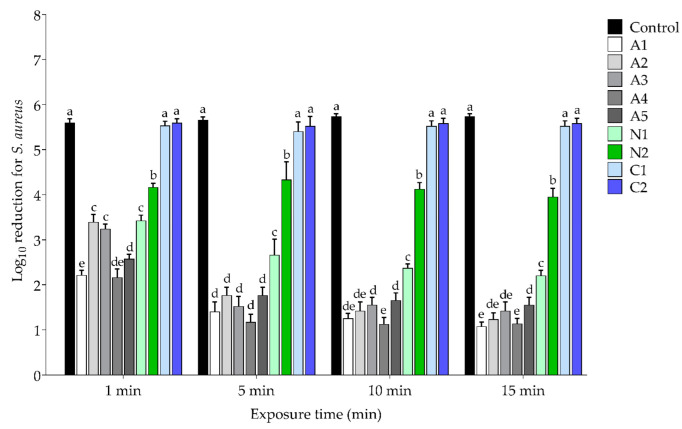
Log_10_ reduction of *S. aureus* at different exposure times to a mixture of CHG and selected lubricating gels. Data represent the mean ± SD of three independent experiments, and different letters (a–e) indicate a significant difference (*p* < 0.05).

**Figure 5:**
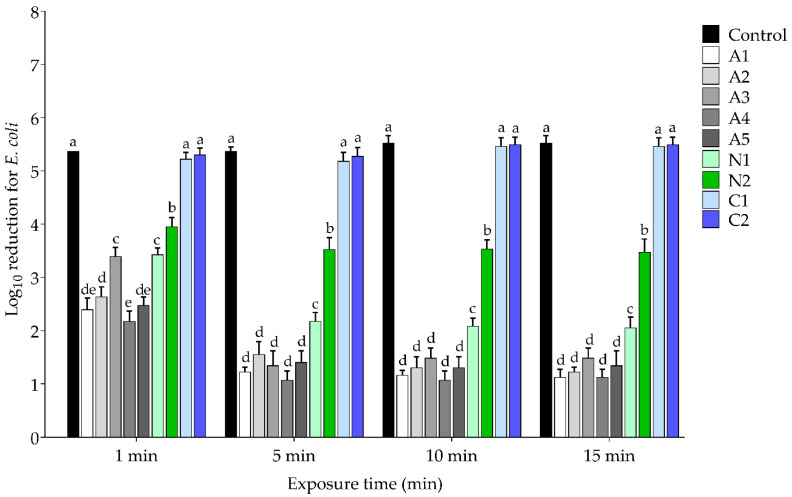
Log_10_ reduction of *E. coli* with different exposure times to a mixture of CHG and selected lubricating gels. Data represent the mean ± SD of three independent experiments, and different letters (a–e) indicate a significant difference (*p* < 0.05).

*S. aureus* and *E. coli* are the predominant bacteria associated with hospital-acquired infections (HAIs); therefore, they were selected to determine the antibacterial activity of 1% CHG after exposure to the lubricating gels and were represented as Gram-positive and Gram-negative bacteria, respectively. The antibacterial activity was assessed at 1-, 5-, 10-, and 15-min exposure times, as shown in Figure 4 and Figure 5 for *S. aureus* and *E. coli*, respectively. The results show that 1% CHG exposed to C1 and C2 cationic lubricating gel achieved the significantly highest (*p* < 0.05) log reduction in both *S. aureus* ATCC25923 and *E. coli* ATCC25922 for all exposure times, with 5.40–5.59 and 5.18–5.49 log reductions, respectively, and these did not significantly differ (*p* > 0.05) when compared with the control. At the same time, the antibacterial activity of CHG after being exposed to all anionic lubricating gels (A1–A5) was significantly reduced (*p* < 0.05) to a 1.07 to 3.39 log reduction for *S. aureus* ATCC25923 and a 1.07 to 3.39 log reduction for *E. coli* ATCC25922 for all exposure times. Similar to the CHG, which was exposed to nonionic lubricating gels (N1–N2) for 1 to 15 min, the log reductions were also significantly reduced (*p* < 0.05) to 2.20-4.16 and 2.05-3.95 for *S. aureus* ATCC25923 and *E. coli* ATCC25922, respectively.

## 4. Discussion

HAIs are nosocomially acquired infections that are not present or incubating at the time of admission to a hospital [25], and they constitute a problem in hospitals worldwide [26,27]. The most prevalent types of HAIs are CAUTI, followed by surgical site infections, primary BSIs, hospital-acquired pneumonia (HAP), VAP, and *Clostridium difficile* infections [28,29]. The main bacteria associated with HAIs are *S. aureus*, *E. coli*, *K. pneumoniae*, *Streptococcus pneumoniae*, *P. aeruginosa*, *A. baumannii*, and *Enterococci* spp. [30,31,32]. These bacteria have a considerable intrinsic resistance and a wide-ranging capacity to develop a multi-drug resistance. The World Health Organization (WHO) classifies them as posing an urgent threat to public health and as requiring the development of novel antibiotics [33]. Recently, a national survey of the status of hospital infection prevention practices in Thailand showed that only 31% of Thai hospitals reported excellent leadership support for infection control [34], and 48% of surveyed practices showed significantly increased rates of CLABSI, CAUTIs, and VAP compared with the survey results in 2014 [35]. An epidermal study of CAUTIs conducted at Maharaj Nakorn Chiang Mai Hospital in northern Thailand showed that the incidence of CAUTIs was 2.37 to 7.83 per 1000 catheter-days; the most common isolated pathogens were *E. coli*, *K. pneumoniae*, *A. baumannii*, *P. aeruginosa*, and *Enterococcus faecium*. Additionally, 36.7% of CAUTI subjects died, and 20.0% of deaths were related to CAUTIs [36]. 

Various formulations and concentrations of disinfectants and antiseptics have been employed on both animate and inanimate surfaces in hospitals [37,38]. Some pathogens are becoming more resistant to disinfectants and antiseptics, leading to the development of novel preparations and various strategies to achieve the maximum efficacy and effectiveness of disinfectants and antiseptics [37,39,40]. CHG is one of the top suggestions among current disinfectants [2]. It constitutes a broad-spectrum antimicrobial agent against Gram-positive and Gram-negative bacteria and fungi that operates by disrupting microbial cell membranes [41]. The positive charge of the chlorhexidine molecule reacts with the negative charge of the phosphate groups on the microbial cell surfaces, causing the cell’s integrity to be damaged, allowing the entry of CHG and the leakage of intracellular components, and ultimately leading to cell death [2], which specifically depends on the CHG concentration. Chlorhexidine (CHX) is present in various forms, and the solubility depends on its salt forms. CHX has solubility at 0.008% *w*/*v* in water at 20 °C, while salt solubility ranges from 0.01 to 70%. CHX is commercially available as CHX digluconate, which has more than 70% solubility in water and is also available at a concentration of 20% *w*/*v* [42]. The aqueous solution of CHX is stable at pH 5 to 8; therefore, a pH higher than 8 and the over-concentration of its solubility can cause the precipitation of CHX. Therefore, understanding the inactivation capacity of CHX is important, as it maintains the antimicrobial activity in the initial setup of this chemical reservoir. This comprises the key to maintaining the CHG concentration required to exert antimicrobial activity. The anionic thickening agent commonly used in alcohol hand sanitizing gels and lubricating gels can deactivate CHG’s persistent activity. 

*E. coli* is a Gram-negative bacteria with a high resistance to ampicillin, levofloxacin, cotrimoxazole, gentamicin, and ciprofloxacin, and *S. aureus* is a Gram-positive bacteria highly resistant to penicillin and ampicillin [43]; therefore, the bacteria were used as control strains to evaluate the in vitro antibacterial activity of CHG and compare it with the mixtures of CHG and collected lubricating gels, thus determining the pharmaceutical incompatibility of eight widely used Thai-marketed lubricating gels in northern Thai hospitals and a lubricating gel formulated with a CHG solution. The pharmaceutical incompatibilities of the lubricating gels containing anionic thickener (A1–A5) and nonionic thickener (N1), and CHG, could be seen both with the naked eye and via HPLC determination. The reduction in CHG might have been due to the interaction between CHG gluconate and the gelling agents. Several publications reported a reduced CHG effectiveness after applying topical agents [15]. Kaiser and coworkers [5] reported significantly reduced CHG resulting from incompatible alcohol hand sanitizing gels, especially in products containing anionic thickening agents, namely, carbomer, in which the 3 log reductions of used indicator bacteria were lower than those of the control. This means that alcohol-based hand sanitizing gels containing carbomer can significantly reduce the antiseptic effectiveness of CHG by a factor of 1000. The interaction between CHX and EDTA can also reduce CHG effectiveness. Combined CHG and EDTA produce a white precipitate. A study by Rasimick et al. [44] on determining the precipitate formed when CHG is mixed with EDTA demonstrated that over 90% of the precipitate was composed of CHX and EDTA, with the precipitation ratio at approximately 1.6 to 1. Occasionally, this incompatibility cannot be observed via visible signs because of the formation of micelles [43]. CHG forms small aggregates in an aqueous solution, and this formation influences the resulting concentration of CHG in the solution [42,45]. Our results showed no incompatibility of CHG with nonionic lubricating gel N2, as determined by the naked eye. However, the results related to chemical and therapeutic incompatibilities showed that the amount of CHG was reduced, and this resulted in a decrease in the disinfection effectiveness in both tested pathogens. Remarkably, a correlation was found between reduced CHG and the antibacterial activity against *S. aureus* and *E. coli*, as shown in Table S1. The obtained results suggest that the effectiveness of CHG residue on the skin can be greatly diminished by the incompatibility of anionic- and nonionic-based gels. Moreover, the sample N2 that was exposed to CHG was analyzed for micelle formation using dynamic light scattering (Zetasizer ZS, Malvern Panalytical Ltd., Malvern, UK). The results show the occurrence of micelle particles with diameters of 98.50 ± 10.16 and 12.68 ± 1.43 nm (Figure S1).

This study demonstrated the possibility of pharmaceutical incompatibility using lubricating gels with CHG, which could reduce the disinfection efficiency. Therefore, our work can be used cautiously to inform several guidelines, especially regarding the urinary catheterization process, wherein a urethral lubricant product is used after cleaning with a CHG solution. Additionally, hand hygiene guidelines include CHG cleansing followed by alcohol hand gel, which may reduce the antimicrobial effect of CHG, even with gel containing anionic and nonionic thickeners. Several guidelines recommend using an alcohol hand rub instead. The incompatibility may result in an increased risk of cross-contamination and cross-infection, especially in countries in the equatorial region where the temperature is favorable to the growth of pathogens. To reduce the rate of cross-infection resulting from the pharmaceutical incompatibility of CHG with anionic- and nonionic-based lubricating gel or alcohol hand sanitizing gel, we need to develop strategies to put the best practices for urinary catheterization and hand hygiene into action. In addition, providing healthcare workers in northern Thailand with a multidisciplinary team consisting of healthcare professionals from different fields, especially infectious disease doctors, infectious disease pharmacists and infectious nurses, will ensure that all staff understand the reasons for changes and agree with the needed changes. 

Although the study provided the in vitro pharmaceutical incompatibility of lubricating gels and CHG, we cannot provide the actual final concentration of the gelling agent in lubricating gels because it is not mentioned on the product’s label, so we do not know the minimum gelling agent that can interact with CHG. However, the recommended concentration of CHG and a proper amount of lubricating gels were used. To break the limitation, a clinical study of CAUTIs’ incidence rate in hospitals using CHG cleaning followed by various cationic lubricating gels for urinary catheterization compared to anionic or nonionic lubricating gels must be performed to confirm the incompatibilities of CHG. 

## 5. Conclusions

In conclusion, these evaluations of the pharmaceutical incompatibility between CHG and lubricating gels from our study can be used cautiously to apply topical products containing anionic gelling agents, anionic surfactants, and other anionic substances that are incompatible with CHG, in order to maintain a persistent antibacterial activity. Moreover, anionic gelling agents are detectable with the naked eye, corresponding to chemical and therapeutic incompatibilities, similar to cationic gelling agents. In contrast, some nonionic gelling agents may show a physical incompatibility that cannot be detected by the naked eye. Therefore, chemical and therapeutic assessments may be required, as micelles may not be visible to the naked eye. 

## Figures and Tables

**Table 1:** Type of gelling agents/thickeners in lubricating gel samples.

No.	Sample	Gelling Agents/Thickeners
1	A1	Acrylates/C10-30 Alkyl Acrylate Cross-polymer
2	A2	Carbopol 941
3	A3	Carbopol 940
4	A4	Sodium acrylate polymers
5	A5	Carbopol 940
6	N1	Hydroxypropyl methylcellulose (HPMC)
7	N2	Hydroxyethyl cellulose (HEC)
8	C1	Positively-charged polysaccharide
9	C2	Positively-charged polysaccharide

## Data Availability

The original contributions generated for this study are included in the article; the data presented in this study are available on request from the corresponding author.

## Supplementary Materials

The following supporting information can be downloaded at: https://www.mdpi.com/article/10.3390/ijerph191912285/s1, Figure S1: The particle size distribution from the dynamic light scattering of (A) CHG, (B) lubricant gel N2 and (C) lubricant gel N2 exposure to CHG; Table S1: Correlation coefficients (r2) between type of lubricating gel/percent reduction of CHG on log reduction of *S. aureus* and *E. coli*.
