# Supplementary material for: Pharmaceutical Incompatibility of Lubricating Gel Formulation Reduces Antibacterial Activity of Chlorhexidine Gluconate: In Vitro Study in Northern Thailand
Source: Int J Environ Res Public Health. 2022 Sep 27;19(19):12285. doi: 10.3390/ijerph191912285 (PMC9566729; doi:10.3390/ijerph191912285)
Supplement: Supplementary file 1 [file ijerph-19-12285-s001.zip › ijerph-1871208-supplementary.pdf]

Supplementary Material

Table S1. Correlation coefficients (r²) between Type of lubricating gel/Percent reduction of CHG on Log reduction of *S. aureus* and *E. coli*.

		S. aureus				*E. coli*			
		**1 min**	**5 min**	**10 min**	**15 min**	**1 min**	**5 min**	**10 min**	**15 min**
**Type of lubricating gel**		0.824 **	0.886 **	0.892 **	0.898 **	0.828 **	0.869 **	0.869 **	0.874 **
**Percent reduction of CHG**	**Exposure time**								
	**1 min**	−0.870 **				−0.836 **			
	**5 min**		−0.948 **				−0.928 **		
	**10 min**			−0.938 **				−0.917 **	
	**15 min**				−0.930				−0.915 **

**. Correlation is significant at the 0.01 level (2-tailed).

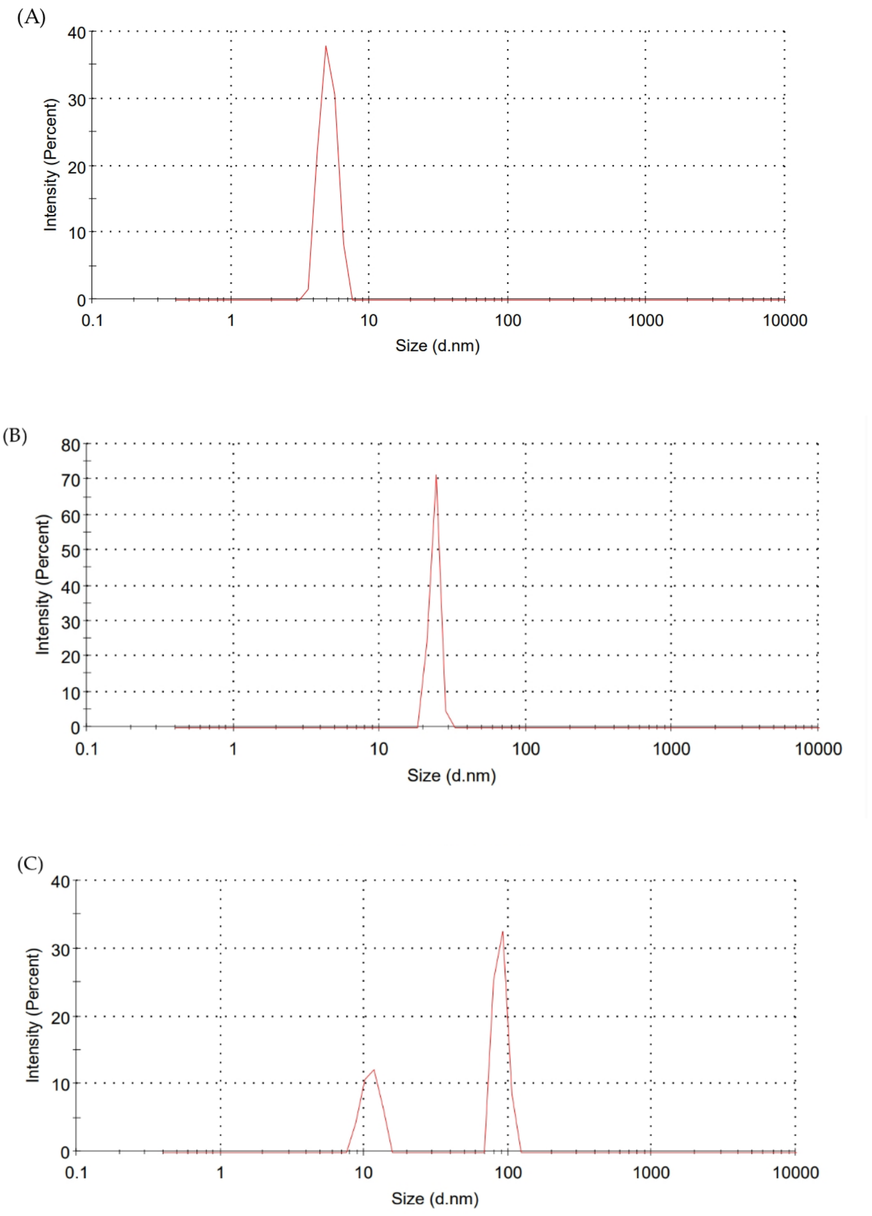

Figure S1. The particle size distribution from the dynamic light scattering of (**A**) CHG, (**B**) lubricant gel N2 and (**C**) lubricant gel N2 exposure to CHG.
